# The Journal of Pathology and Bacteriology

**Published:** 1892-12

**Authors:** 


*The Journal of Pathology and Bacteriology.* Edited by GERMAN SIMS WOODHEAD, M.D. Vol. I. Nos. I. and II. Edinburgh: YOUNG J. PENTLAND. 1892.

There has long been felt the want of a publication containing reports of experimental and bacteriological research in pathology, and which shall appeal to a larger circle of readers, and be of more general interest than are the proceedings of the Pathological Society.

This *Journal*, which we owe to the energy of Dr. Woodhead, is intended to supply such a want. If the succeeding numbers come up to the level of these, we shall soon escape the reproach that English pathologists do but little towards the advancement of a science on which the progress of practical medicine and surgery so largely depends.

It is obviously impossible to give even a sketch of the more important questions elucidated by the various papers in these numbers; but we may perhaps be permitted to glance at the results of two or three of the researches here published. And we may first take Mr. Percy Dean's investigations on localised intracranial pressure. He shows that a spreading œdema is set up by any body which compresses the brain sufficiently to give rise to circulatory disturbance, and that this œdema continues to spread after the removal of the body, so as to cause death. This is paralleled by many recorded instances, in which removal of a bloodclot, due to meningeal hemorrhage, has been followed, though no more bleeding occur, by progressive weakness and insensibility with fever, and death within twenty-four hours. Mr. Dean shows that, by opening up the wound afresh, letting the brain bulge freely into the trephine hole, and, if necessary, freely incising it, the œdema can be prevented from spreading, and the patient saved. Mr. Dean's researches also explain and correlate two well-known results of unilateral cerebral hemorrhage. We owe to Dr. Bastian the knowledge that whilst the hemorrhage is, as in-indicated by the symptoms, small in amount, the surface temperature on the same side of the body is lower than on the opposite (paralysed) side; whilst, if the bleeding continue, the conditions become exactly reversed. Mr. Hutchinson has shown that, in cases of cerebral hemorrhage, the pupil on the same side is at first very small; but as the bleeding continues, it becomes dilated and immovable (the well-known "Hutchinson pupil"). Mr. Dean's researches explain these clinical observations; they show that if the compressing body be small, an irritative lesion is produced, causing an elevation of the surface temperature of the opposite (paralysed) side, and a contraction of the pupil of the same side; whilst if the body be large, a paralytic lesion is produced, causing a depression of the temperature of the opposite side and dilatation of the pupil of the same side.

Although we have already taken up so much space, we must not omit to call attention to Dr. Herbert Spencer's paper, in which it is shown that the localised induration in the sterno-mastoid muscle of new-born children is due to a hæmatoma. Mention must also be made of the beautiful illustrations accompanying the paper by Drs. Ruffer and Walker on parasitic protozoa in cancer.

Such researches as these thoroughly justify the opinion expressed above, that experimental pathological work by Englishmen will materially advance the progress of clinical medicine and surgery, and that the establishment of this periodical will do much to further this good work.

The *Journal* is printed in the most admirable manner, and the illustrations throughout are worthy of the highest praise.

